# (1*S*,3*R*,8*R*,9*S*,11*R*)-2,2,10,10-Tetra­chloro-3,7,7,11-tetra­methyl­tetra­cyclo­[6.5.0.0^1,3^.0^9,11^]trideca­ne

**DOI:** 10.1107/S1600536813001700

**Published:** 2013-01-23

**Authors:** Najia Ourhriss, Ahmed Benharref, Mohamed Saadi, Lahcen El Ammari, Moha Berraho

**Affiliations:** a Laboratoire de Chimie Biomoléculaire, Substances Naturelles et Réactivité "Unité Associée au CNRST(URAC16)", Faculté des Sciences Semlalia, BP 2390, Bd My Abdellah, 40000 Marrakech, Morocco; b Laboratoire de Chimie du Solide Appliquée, Faculté des Sciences, Avenue Ibn Battouta, BP 1014 Rabat, Morocco

## Abstract

The title compound, C_17_H_24_Cl_4_, was synthesized from β-himachalene (3,5,5,9-tetra­methyl-2,4a,5,6,7,8-hexa­hydro-1*H*-benzocyclo­heptene), which was isolated from the essential oil of the Atlas cedar (*Cedrus Atlantica*). The mol­ecule is built up from fused six- and seven-membered rings and two three-membered rings from the reaction of β-himachalene with dichloro­carbene. The six-membered ring shows a chair conformation, whereas the seven-membered ring displays a boat conformation.

## Related literature

For the isolation of β-himachalene, see: Joseph & Dev (1968 ▶); Plattier & Teisseire (1974 ▶). For the reactivity of this sesquiterpene, see: Lassaba *et al.* (1998 ▶); Chekroun *et al.* (2000 ▶); El Jamili *et al.* (2002 ▶); Sbai *et al.* (2002 ▶); Dakir *et al.* (2004 ▶). For its biological activity, see: Daoubi *et al.* (2004 ▶). For puckering parameters, see: Cremer & Pople (1975 ▶).
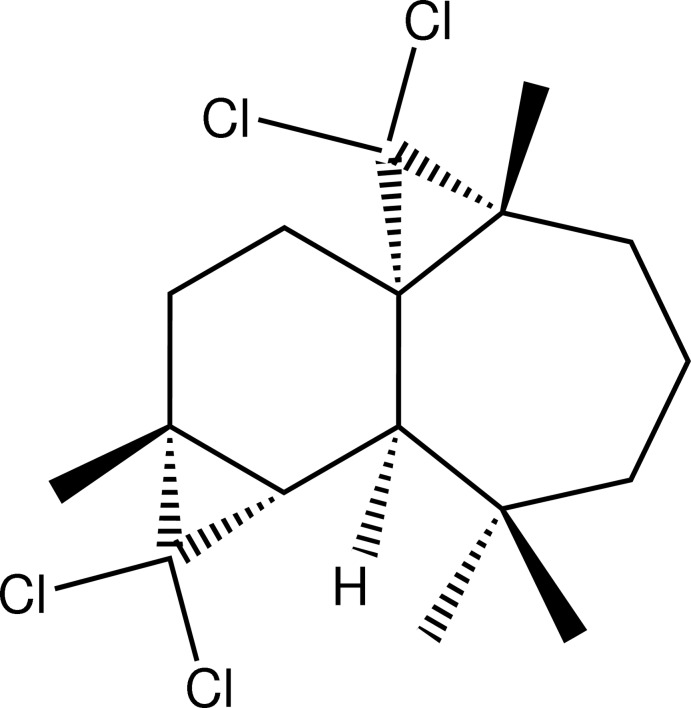


## Experimental

### 

#### Crystal data

C_17_H_24_Cl_4_
*M* *_r_* = 370.16
Monoclinic, 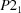

*a* = 8.8807 (6) Å
*b* = 11.6280 (8) Å
*c* = 9.0596 (6) Å
β = 107.665 (2)°
*V* = 891.42 (10) Å^3^
*Z* = 2
Mo *K*α radiation
μ = 0.66 mm^−1^
*T* = 296 K
0.41 × 0.35 × 0.27 mm

#### Data collection

Bruker X8 APEX diffractometer
15112 measured reflections
5419 independent reflections
4910 reflections with *I* > 2σ(*I*)
*R*_int_ = 0.019

#### Refinement

*R*[*F* ^2^ > 2σ(*F* ^2^)] = 0.032
*wR*(*F* ^2^) = 0.085
*S* = 1.03
5419 reflections
190 parameters
1 restraint
H-atom parameters constrained
Δρ_max_ = 0.32 e Å^−3^
Δρ_min_ = −0.19 e Å^−3^
Absolute structure: Flack (1983 ▶)
Flack parameter: 0.04 (4)

### 

Data collection: *APEX2* (Bruker, 2009 ▶); cell refinement: *SAINT* (Bruker, 2009 ▶); data reduction: *SAINT*; program(s) used to solve structure: *SHELXS97* (Sheldrick, 2008 ▶); program(s) used to refine structure: *SHELXL97* (Sheldrick, 2008 ▶); molecular graphics: *ORTEP-3 for Windows* (Farrugia, 2012 ▶); software used to prepare material for publication: *PLATON* (Spek, 2009 ▶) and *publCIF* (Westrip, 2010 ▶).

## Supplementary Material

Crystal structure: contains datablock(s) I, global. DOI: 10.1107/S1600536813001700/bt6883sup1.cif

Structure factors: contains datablock(s) I. DOI: 10.1107/S1600536813001700/bt6883Isup2.hkl

Supplementary material file. DOI: 10.1107/S1600536813001700/bt6883Isup3.cml

Additional supplementary materials:  crystallographic information; 3D view; checkCIF report

## Figures and Tables

**Fig. 1.**
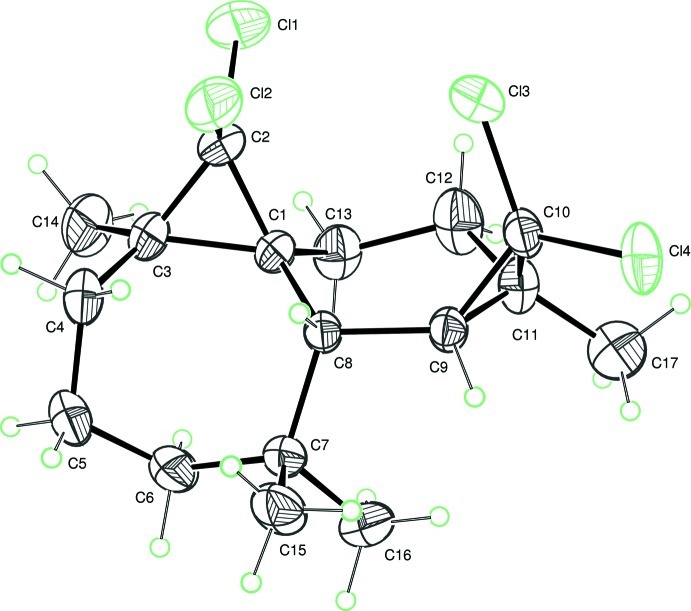
: Molecular structure of the title compound with the atom-labelling scheme. Displacement ellipsoids are drawn at the 50% probability. level. H atoms are represented as small spheres of arbitrary radii.

## supplementary crystallographic information

### Comment

The bicyclic sesquiterpene, β-himachalene is the main constituent (50%) of the essential oil of the Atlas cedar (Cedrus atlantica) (Joseph & Dev, 1968; Plattier & Teisseire, 1974). The reactivity of this sesquiterpene and its derivatives has been studied extensively by our team in order to prepare new products having biological proprieties (Lassaba *et al.*, 1998; Chekroun *et al.*, 2000; El Jamili *et al.*, 2002; Sbai *et al.*, 2002; Dakir *et al.*, 2004). Indeed, these compounds were tested, using the food poisoning technique, for their potential antifungal activity against phytopathogen Botrytis cinerea (Daoubi *et al.*, 2004). Thus the action of two equivalents of dichlorocarbene, generated *in situ* from chloroform in the presence of sodium hydroxide as base and n-benzyltriethylammonium chloride as catalyst, on β-himachalene leads to a mixture of two diastereisomers: (1*S*,3*R*,8*R*,9S,11*R*)-2,2,10,10-tetrachloro-3,7,7,11-tetraméthyletetracyclo [6,5,0,01.2,09.11] tridecane (*X*) and its isomer (1*S*,3*R*,8*R*,9*R*,11*S*)-2,2,10,10- tetrachloro-3,7,7,11-tetraméthyletetracyclo [6,5,0,01.2,09.11]tridecane (Y), in an over-all yield of 80% and 85/15 ratio. By single-crystal X-ray diffraction analysis, we have determined the absolute configuration of *X* and we deduced that from its isomer Y.

The molecule contains a fused six- and seven-membered rings, which is fused to two three-membered rings as schown in Fig. 1. The six-membered ring has a chair conformation, with as indicated by the total puckering amplitude QT = 0.4518 (19) Å and spherical polar angle θ = 140.4 (2)° with φ2 = 141.5 (4)°, whereas the seven-membered ring displays a boat conformation with QT = 1.1323 (2) Å, θ2 = 87.3 (1), φ2 = -48.94 (9)° and φ3 = -125 (2)° (Cremer & Pople, 1975). The dihedral angle between the five- and seven-membered rings is 55.89 (9)°. The three-membered ring (C1, C2, C3) ring is nearly perpendicular to the six-membered ring (C1, C8, C9, C11, C12, C13) with a dihedral angle of 84.24 (19)°. Owing to the presence of Cl atoms, the absolute configuration could be determined from anomalous dispersion effects, by refining the Flack parameter (Flack, 1983) as C1(*S*), C3(*R*), C8(*R*), C9(*S*), and C11(*R*).

### Experimental

A solution containing 6 g (29 mmol) of β-himachalene and 6 mL (75 mmol) of CHCl_3_ in 40 ml of dichloromethane was added dropwise at 273 K over 30 min to 1.6 g (40 mmol) of pulverized sodium hydroxide and 60 mg of *N*–benzyltriethylammonium chloride placed in a 100 ml three–necked flask. After stirring at room temperature for 2 h, the mixture was filtered on celite and concentrated in vacuum. The residue obtained was chromatographed on silicagel column impregnated with silver nitrate (10%) with a mixture of hexane - ethyl acetate (98–2) used as eluent. The two diastereoisomers (1*S*,3*R*,8*R*,9S,11*R*)-2,2,10,10-tetrachloro-3,7,7,11-tetraméthyletetracyclo [6,5,0,01.2,09.11] tridecane (*X*) and (1*S*,3*R*,8*R*,9*R*,11*S*)-2,2,10,10-tetrachloro-3,7,7,11-tetraméthyletetracyclo [6,5,0,01.2,09.11]tridecane (Y), were obtained by this procedure in a 85/15 ratio and a combined yield of 80% (8,5 g; 23,2 mmol). The title compound (isomer *X*) was recrystallized from pentane.

### Refinement

All H atoms were fixed geometrically and treated as riding with C—H = 0.96 Å (methyl),0.97 Å (methylene), 0.98 Å (methine) with *U*_iso_(H) = 1.2Ueq (methylene, methine) or *U*_iso_(H) = 1.5Ueq (methyl). The space group is not centrosymmetric and the polar axis restraint is generated automatically by the *SHELXL* program. Friedel pairs were not merged.

### Crystal data

**Table:**

C_17_H_24_Cl_4_	*F*(000) = 388
*M**_r_* = 370.16	*D*_x_ = 1.379 Mg m^−^^3^
Monoclinic, *P*2_1_	Mo *K*α radiation, λ = 0.71073 Å
Hall symbol: P 2yb	Cell parameters from 5419 reflections
*a* = 8.8807 (6) Å	θ = 2.8–30.5°
*b* = 11.6280 (8) Å	µ = 0.66 mm^−^^1^
*c* = 9.0596 (6) Å	*T* = 296 K
β = 107.665 (2)°	Block, colourless
*V* = 891.42 (10) Å^3^	0.41 × 0.35 × 0.27 mm
*Z* = 2	

### Data collection

**Table:**

Bruker X8 APEX diffractometer	4910 reflections with *I* > 2σ(*I*)
Radiation source: fine-focus sealed tube	*R*_int_ = 0.019
Graphite monochromator	θ_max_ = 30.5°, θ_min_ = 2.8°
φ and ω scans	*h* = −12→12
15112 measured reflections	*k* = −16→16
5419 independent reflections	*l* = −12→12

### Refinement

**Table:**

Refinement on *F*^2^	Secondary atom site location: difference Fourier map
Least-squares matrix: full	Hydrogen site location: inferred from neighbouring sites
*R*[*F*^2^ > 2σ(*F*^2^)] = 0.032	H-atom parameters constrained
*wR*(*F*^2^) = 0.085	*w* = 1/[σ^2^(*F*_o_^2^) + (0.0491*P*)^2^ + 0.074*P*] where *P* = (*F*_o_^2^ + 2*F*_c_^2^)/3
*S* = 1.03	(Δ/σ)_max_ = 0.001
5419 reflections	Δρ_max_ = 0.32 e Å^−^^3^
190 parameters	Δρ_min_ = −0.19 e Å^−^^3^
1 restraint	Absolute structure: Flack (1983)
Primary atom site location: structure-invariant direct methods	Flack parameter: 0.04 (4)

### Special details

**Table:**

Geometry. All e.s.d.'s (except the e.s.d. in the dihedral angle between two l.s. planes) are estimated using the full covariance matrix. The cell e.s.d.'s are taken into account individually in the estimation of e.s.d.'s in distances, angles and torsion angles; correlations between e.s.d.'s in cell parameters are only used when they are defined by crystal symmetry. An approximate (isotropic) treatment of cell e.s.d.'s is used for estimating e.s.d.'s involving l.s. planes.
Refinement. Refinement of *F*^2^ against ALL reflections. The weighted *R*-factor *wR* and goodness of fit *S* are based on *F*^2^, conventional *R*-factors *R* are based on *F*, with *F* set to zero for negative *F*^2^. The threshold expression of *F*^2^ > σ(*F*^2^) is used only for calculating *R*-factors(gt) *etc*. and is not relevant to the choice of reflections for refinement. *R*-factors based on *F*^2^ are statistically about twice as large as those based on *F*, and *R*- factors based on ALL data will be even larger.

### Fractional atomic coordinates and isotropic or equivalent isotropic displacement parameters (Å^2^)

**Table:**

	*x*	*y*	*z*	*U*_iso_*/*U*_eq_	
Cl1	0.38317 (6)	0.32772 (6)	0.92294 (6)	0.06701 (17)	
Cl2	0.52164 (6)	0.12723 (5)	0.83384 (6)	0.05443 (13)	
Cl3	0.78628 (6)	0.23092 (5)	1.13768 (5)	0.05374 (13)	
Cl4	1.12169 (5)	0.25246 (4)	1.19453 (5)	0.04960 (12)	
C1	0.62379 (15)	0.35612 (13)	0.77816 (16)	0.0288 (3)	
C2	0.49711 (18)	0.27743 (16)	0.80650 (18)	0.0391 (4)	
C3	0.47133 (16)	0.32604 (17)	0.64690 (18)	0.0380 (3)	
C4	0.48758 (19)	0.24243 (18)	0.52377 (18)	0.0431 (4)	
H4A	0.5552	0.1791	0.5733	0.052*	
H4B	0.3845	0.2111	0.4692	0.052*	
C5	0.5566 (2)	0.2997 (2)	0.40815 (19)	0.0518 (5)	
H5A	0.4729	0.3396	0.3311	0.062*	
H5B	0.5980	0.2408	0.3552	0.062*	
C6	0.6879 (2)	0.38477 (18)	0.48244 (18)	0.0442 (4)	
H6A	0.6393	0.4506	0.5156	0.053*	
H6B	0.7306	0.4116	0.4019	0.053*	
C7	0.82871 (16)	0.34716 (14)	0.62113 (16)	0.0324 (3)	
C8	0.77955 (14)	0.30517 (11)	0.76794 (14)	0.0244 (2)	
H8	0.7717	0.2249	0.7579	0.029*	
C9	0.91820 (15)	0.32805 (12)	0.91322 (15)	0.0269 (2)	
H9	1.0184	0.3143	0.8884	0.032*	
C10	0.93290 (17)	0.29978 (13)	1.07857 (15)	0.0317 (3)	
C11	0.91735 (18)	0.42382 (14)	1.02843 (17)	0.0350 (3)	
C12	0.7581 (2)	0.48145 (17)	1.0110 (2)	0.0474 (4)	
H12A	0.7766	0.5613	1.0420	0.057*	
H12B	0.7095	0.4446	1.0812	0.057*	
C13	0.64262 (19)	0.47670 (14)	0.84681 (19)	0.0377 (3)	
H13A	0.5402	0.5044	0.8488	0.045*	
H13B	0.6798	0.5277	0.7806	0.045*	
C14	0.3446 (2)	0.4161 (2)	0.5839 (2)	0.0545 (5)	
H14A	0.3648	0.4816	0.6516	0.082*	
H14B	0.2430	0.3844	0.5775	0.082*	
H14C	0.3457	0.4395	0.4826	0.082*	
C15	0.9169 (2)	0.2471 (2)	0.57360 (19)	0.0469 (4)	
H15A	0.8513	0.1798	0.5550	0.070*	
H15B	1.0128	0.2318	0.6552	0.070*	
H15C	0.9418	0.2672	0.4809	0.070*	
C16	0.9411 (2)	0.45147 (18)	0.6573 (2)	0.0459 (4)	
H16A	1.0290	0.4353	0.7471	0.069*	
H16B	0.8854	0.5177	0.6767	0.069*	
H16C	0.9789	0.4664	0.5704	0.069*	
C17	1.0594 (2)	0.50220 (16)	1.0855 (2)	0.0529 (5)	
H17A	1.0320	0.5781	1.0443	0.079*	
H17B	1.1446	0.4729	1.0518	0.079*	
H17C	1.0917	0.5053	1.1967	0.079*	

### Atomic displacement parameters (Å^2^)

**Table:**

	*U*^11^	*U*^22^	*U*^33^	*U*^12^	*U*^13^	*U*^23^
Cl1	0.0372 (2)	0.1166 (5)	0.0572 (3)	0.0018 (3)	0.0294 (2)	−0.0039 (3)
Cl2	0.0510 (2)	0.0575 (3)	0.0527 (3)	−0.0213 (2)	0.0126 (2)	0.0075 (2)
Cl3	0.0529 (2)	0.0783 (3)	0.03199 (18)	−0.0149 (2)	0.01578 (16)	0.00474 (19)
Cl4	0.0432 (2)	0.0512 (2)	0.03845 (19)	0.00492 (18)	−0.01151 (15)	0.00149 (18)
C1	0.0203 (5)	0.0383 (7)	0.0276 (6)	0.0004 (5)	0.0068 (5)	−0.0003 (5)
C2	0.0253 (6)	0.0579 (10)	0.0358 (7)	−0.0060 (6)	0.0120 (5)	−0.0007 (7)
C3	0.0202 (6)	0.0576 (10)	0.0339 (7)	−0.0013 (6)	0.0046 (5)	0.0000 (7)
C4	0.0305 (7)	0.0607 (10)	0.0318 (7)	−0.0065 (7)	−0.0001 (5)	−0.0055 (7)
C5	0.0408 (8)	0.0849 (15)	0.0247 (7)	−0.0039 (9)	0.0024 (6)	−0.0019 (8)
C6	0.0364 (8)	0.0666 (12)	0.0288 (7)	0.0003 (7)	0.0089 (6)	0.0111 (7)
C7	0.0268 (6)	0.0455 (8)	0.0260 (6)	−0.0005 (6)	0.0095 (5)	0.0001 (6)
C8	0.0209 (5)	0.0299 (6)	0.0212 (5)	0.0004 (5)	0.0049 (4)	−0.0022 (5)
C9	0.0220 (5)	0.0327 (6)	0.0244 (6)	0.0006 (5)	0.0048 (4)	−0.0024 (5)
C10	0.0295 (6)	0.0385 (7)	0.0234 (6)	0.0007 (6)	0.0023 (5)	−0.0018 (5)
C11	0.0321 (7)	0.0350 (7)	0.0317 (7)	0.0009 (6)	0.0006 (5)	−0.0070 (6)
C12	0.0456 (9)	0.0486 (9)	0.0434 (9)	0.0131 (7)	0.0066 (7)	−0.0172 (7)
C13	0.0319 (7)	0.0384 (8)	0.0412 (8)	0.0100 (6)	0.0087 (6)	−0.0023 (6)
C14	0.0264 (7)	0.0793 (14)	0.0513 (10)	0.0126 (8)	0.0020 (7)	0.0039 (10)
C15	0.0433 (8)	0.0676 (11)	0.0341 (7)	0.0100 (8)	0.0184 (6)	−0.0059 (8)
C16	0.0372 (8)	0.0578 (11)	0.0453 (9)	−0.0095 (7)	0.0162 (7)	0.0063 (8)
C17	0.0513 (10)	0.0410 (9)	0.0527 (10)	−0.0117 (8)	−0.0050 (8)	−0.0085 (8)

### Geometric parameters (Å, º)

**Table:**

Cl1—C2	1.7682 (17)	C8—H8	0.9380
Cl2—C2	1.768 (2)	C9—C10	1.5001 (19)
Cl3—C10	1.7454 (16)	C9—C11	1.528 (2)
Cl4—C10	1.7744 (14)	C9—H9	0.9951
C1—C13	1.522 (2)	C10—C11	1.506 (2)
C1—C2	1.531 (2)	C11—C17	1.515 (2)
C1—C8	1.5332 (17)	C11—C12	1.529 (2)
C1—C3	1.5468 (19)	C12—C13	1.530 (2)
C2—C3	1.504 (2)	C12—H12A	0.9700
C3—C14	1.517 (2)	C12—H12B	0.9700
C3—C4	1.519 (2)	C13—H13A	0.9700
C4—C5	1.519 (3)	C13—H13B	0.9700
C4—H4A	0.9700	C14—H14A	0.9600
C4—H4B	0.9700	C14—H14B	0.9600
C5—C6	1.521 (3)	C14—H14C	0.9600
C5—H5A	0.9700	C15—H15A	0.9600
C5—H5B	0.9700	C15—H15B	0.9600
C6—C7	1.543 (2)	C15—H15C	0.9600
C6—H6A	0.9700	C16—H16A	0.9600
C6—H6B	0.9700	C16—H16B	0.9600
C7—C15	1.536 (2)	C16—H16C	0.9600
C7—C16	1.541 (2)	C17—H17A	0.9600
C7—C8	1.5970 (19)	C17—H17B	0.9600
C8—C9	1.5283 (17)	C17—H17C	0.9600
			
C13—C1—C2	118.50 (12)	C10—C9—H9	112.2
C13—C1—C8	113.04 (11)	C11—C9—H9	117.5
C2—C1—C8	120.10 (13)	C8—C9—H9	108.6
C13—C1—C3	118.97 (13)	C9—C10—C11	61.10 (10)
C2—C1—C3	58.50 (10)	C9—C10—Cl3	124.05 (10)
C8—C1—C3	117.48 (12)	C11—C10—Cl3	121.55 (11)
C3—C2—C1	61.28 (10)	C9—C10—Cl4	116.07 (10)
C3—C2—Cl2	118.84 (13)	C11—C10—Cl4	117.34 (11)
C1—C2—Cl2	123.25 (12)	Cl3—C10—Cl4	109.57 (8)
C3—C2—Cl1	120.10 (13)	C10—C11—C17	118.86 (14)
C1—C2—Cl1	119.01 (12)	C10—C11—C9	59.27 (9)
Cl2—C2—Cl1	108.16 (9)	C17—C11—C9	119.72 (15)
C2—C3—C14	120.05 (14)	C10—C11—C12	116.63 (15)
C2—C3—C4	116.41 (16)	C17—C11—C12	114.83 (15)
C14—C3—C4	112.99 (14)	C9—C11—C12	116.42 (12)
C2—C3—C1	60.23 (9)	C11—C12—C13	114.26 (13)
C14—C3—C1	120.64 (16)	C11—C12—H12A	108.7
C4—C3—C1	116.95 (12)	C13—C12—H12A	108.7
C5—C4—C3	111.97 (17)	C11—C12—H12B	108.7
C5—C4—H4A	109.2	C13—C12—H12B	108.7
C3—C4—H4A	109.2	H12A—C12—H12B	107.6
C5—C4—H4B	109.2	C1—C13—C12	112.86 (13)
C3—C4—H4B	109.2	C1—C13—H13A	109.0
H4A—C4—H4B	107.9	C12—C13—H13A	109.0
C4—C5—C6	113.30 (13)	C1—C13—H13B	109.0
C4—C5—H5A	108.9	C12—C13—H13B	109.0
C6—C5—H5A	108.9	H13A—C13—H13B	107.8
C4—C5—H5B	108.9	C3—C14—H14A	109.5
C6—C5—H5B	108.9	C3—C14—H14B	109.5
H5A—C5—H5B	107.7	H14A—C14—H14B	109.5
C5—C6—C7	119.92 (16)	C3—C14—H14C	109.5
C5—C6—H6A	107.3	H14A—C14—H14C	109.5
C7—C6—H6A	107.3	H14B—C14—H14C	109.5
C5—C6—H6B	107.3	C7—C15—H15A	109.5
C7—C6—H6B	107.3	C7—C15—H15B	109.5
H6A—C6—H6B	106.9	H15A—C15—H15B	109.5
C15—C7—C16	107.65 (13)	C7—C15—H15C	109.5
C15—C7—C6	110.06 (13)	H15A—C15—H15C	109.5
C16—C7—C6	105.23 (14)	H15B—C15—H15C	109.5
C15—C7—C8	107.07 (13)	C7—C16—H16A	109.5
C16—C7—C8	112.76 (12)	C7—C16—H16B	109.5
C6—C7—C8	113.95 (11)	H16A—C16—H16B	109.5
C9—C8—C1	112.74 (10)	C7—C16—H16C	109.5
C9—C8—C7	108.15 (10)	H16A—C16—H16C	109.5
C1—C8—C7	114.32 (11)	H16B—C16—H16C	109.5
C9—C8—H8	105.9	C11—C17—H17A	109.5
C1—C8—H8	110.4	C11—C17—H17B	109.5
C7—C8—H8	104.7	H17A—C17—H17B	109.5
C10—C9—C11	59.64 (10)	C11—C17—H17C	109.5
C10—C9—C8	128.69 (12)	H17A—C17—H17C	109.5
C11—C9—C8	123.06 (11)	H17B—C17—H17C	109.5
